# The effects of seaward distance on above and below ground carbon stocks in estuarine mangrove ecosystems

**DOI:** 10.1186/s13021-020-00161-4

**Published:** 2020-12-07

**Authors:** Georgia de Jong Cleyndert, Aida Cuni-Sanchez, Hamidu A. Seki, Deo D. Shirima, Pantaleo K. T. Munishi, Neil Burgess, Kim Calders, Robert Marchant

**Affiliations:** 1 York Institute for Tropical Ecosystems, Department of Environment and Geography, University of York, Heslington, York, North Yorkshire, YO10 5NG UK; 2 Department of Ecosystem Science and Sustainability, Colorado State University, Campus Delivery 1476, Fort Collins, CO 80523 USA; 3 Department of Geography and Economics, Faculty of Humanities and Social Sciences, Mkwawa University College of Education, Iringa, Tanzania; 4 Department of Ecosystems and Conservation, College of Forestry, Wildlife and Tourism, Sokoine University of Agriculture, Morogoro, Tanzania; 5 UNEP-WCMC, 219 Huntington Road, Cambridge, UK; 6 CMEC, GLOBE Institute, University of Copenhagen, Copenhagen, Denmark; 7 National Carbon Monitoring Centre, Sokoine Unversity of Agriculture, Morogoro, Tanzania; 8 CAVElab – Computational & Applied Vegetation Ecology, Department of Environment, Ghent University, Ghent, Belgium

**Keywords:** Africa, Blue Carbon, REDD+, Sediment, Mangrove management

## Abstract

**Background:**

Mangrove forests have gained recognition for their potential role in climate change mitigation due to carbon sequestration in live trees, and carbon storage in the sediments trapped by mangrove tree roots and pneumatophores. Africa hosts about 19% of the world’s mangroves, yet relatively few studies have examined the carbon stocks of African mangroves. The available studies report considerable differences among sites and amongst the different pools of carbon stocks. None considered the effects of seaward distance. We present details of AGC and SOC carbon stocks for Lindi in Tanzania, and focus on how these values differ with increasing seaward distance and, how our results compare to those reported elsewhere across Africa.

**Results:**

AGC ranged between 11 and 55 Mg C ha^−1^, but was not significantly affected by seaward distance. SOC for 0–1 m depth ranged from 154 to 484, with a mean of 302 Mg C ha^−1^. SOC was significantly negatively correlated with seaward distance. Mangrove type (estuarine/oceanic), soil erosion, soil depth may explain these differences We note important methodological differences in previous studies on carbon stocks in mangroves in Africa.

**Conclusion:**

This study indicates that seaward distance has an important effect on SOC stocks in the Lindi region of Tanzania. SOC should be fully incorporated into national climate change mitigation policies. Studies should report seaward distance and to describe the type of mangrove stand to make results easily comparable across sites and to assess the true value of Blue Carbon in Africa. We recommend focusing on trees > 10 cm diameter for AGC, and sampling soils to at least 1 m depth for SOC, which would provide a more complete assessment of the potentially considerable mangrove carbon store.

## Background

Mangroves are salt-tolerant ecosystems that grow at the interface between land and sea in tropical and sub-tropical latitudes [1, 2]. Mangroves provide a number of important ecosystem services to humans; in addition to being an essential source of building materials and firewood, they act as irreplaceable nursery habitats for economically and ecologically valuable marine species [3–5] and provide coastal protection from waves and storms [6, 7]. Additionally, they improve water quality through nutrient recycling and sediment regulation [5, 8]. More recently, mangrove ecosystems have gained recognition for their potential role in climate change mitigation due to the carbon sequestration in trees and storage in the sediments that are trapped by the mangrove tree roots and pneumatophores [8–10]. Together with seagrass beds and salt marshes, mangroves form the ‘Blue Carbon’ ecosystems [11] which are attracting increased attention as one way to store carbon and reduce the speed of global warming. Although coastal vegetated habitats represent a much smaller area than terrestrial forests, their total contribution to long-term carbon sequestration is comparable to carbon sinks in terrestrial ecosystem types [10]. Like many other forests and woodlands, because primary production exceeds respiration, mangroves are net autotrophic ecosystem and produce more energy than they utilise [12, 13] and therefore function, if not degraded, as one of the most effective global CO_2_ sinks [14]. Mangroves have the greatest carbon stock among the Blue Carbon ecosystems, storing 6.5 Pg carbon globally, whilst saltmarshes and sea grass meadows stock 2.0 and 2.3 Pg carbon, respectively [15]. Notwithstanding this potential interest the details of the mangrove carbon store and how this responds to drivers of change remain relatively unknown: of 13,000 peer‐reviewed papers published on mangroves over the past 30 years, less than 1%, most in the last 10 years, examined their role in the carbon cycle [16].

Despite their importance, over the past 60 years more than one-third of the world’s mangroves have been lost [17], but the history of their degradation extends through centuries [18]. Coastal development, aquaculture expansion and overharvesting for boat building (timber and poles), building material and firewood are the primary anthropogenic drivers of loss of mangroves [5, 19–21]. Natural drivers that drive changes in mangrove composition and distribution are also important and include hydrological dynamics, the impacts of extreme weather events and sea-level rise which are projected to increase in frequency and magnitude due to global climate change, respectively [6, 21]. As climate change mitigation has come to the fore of international scientific and political discussions [22], there has been an enhanced focus on conserving and restoring degraded ecosystems that are known to function as carbon sinks [10, 22], through mechanisms such as Reducing Emissions from Deforestation and Degradation (REDD+) and other United Nations Framework Convention on Climate Change (UNFCCC) mechanisms increasingly aim to support livelihood developments and mitigate climate change impacts through Green Climate Fund investments [23]. The significance of ‘blue’ carbon processes, pools and sinks need to be centrally factored into decision making at all scales—from global policy issues on climate change, through to resource management at sectoral (e.g. fisheries) and national levels, and even as a criterion in the selection of prospective Marine Protected Areas [24].

There has been long term interest around “interface” mangrove ecosystems that couple upland terrestrial and coastal ecosystems, with the shift from documenting their zonation and interaction with human use, through to increasing work on their biogeochemical cycling. Alongi and Mukhopadhyay (2015) estimated that low latitude mangrove ecosystems typically store between 100 and 400 tonnes of carbon per hectare; sequestering and releasing more carbon by area than almost any other coastal ecosystem [25]. Africa hosts about 19% of the world’s mangroves, yet there are relatively few studies that have examined the carbon stocks of African mangroves [26], and the studies available report great differences among sites and amongst the different pools of carbon stocks, particularly between the above ground carbon (AGC) stored in the trees and the organic carbon stored within the sediment-‘soil organic carbon (SOC)’. For example, SOC estimates for 1 m depth range from 122 Mg C ha^−1^ in Republic of Congo [27] to 342 Mg C ha^−1^ in Liberia [26]. In a single estuary in Liberia, total ecosystem carbon stocks (AGC + total SOC) varied by over fourfold, ranging from 366 to 1485 Mg C ha^1^ [28].

In mangroves, high SOC is linked with slow decomposition of organic matter due to waterlogged saline environments which impedes microbial degradation [10, 17, 29, 30]. Differences in SOC can be explained by the differences in waterlogging, nutrients and salinity, linked to whether mangroves are classified as oceanic, estuarine, riverine or interior, and also to salinity/nutrient changes related to tidal inundation and seaward distance. Two recent reviews on SOC in mangroves pointed out at the importance of considering hydrogeomorphological processes in distinct coastal environmental settings [31, 32]. In Indonesia, Weiss et al. note the importance of both the relative seaward distance and the knowledge of the oceanic or estuarine nature of the mangrove ecosystem in estimating the SOC stocks [33]. A recent summary of carbon stocks data from published data from 190 mangrove sites showed that lower mean pore water salinity (related to mangrove type and seaward distance) also affects AGC [28], as in less saline environments more carbon is allocated to aboveground biomass than to roots [31]. However, only a few available studies from Africa report the type of mangroves studied, and none mention seaward distance.

Considerable variation in above-ground carbon in mangroves (AGC, the part stored in aerial parts of trees) has been reported for Africa: from 26 C Mg ha^−1^ in Guinea-Bissau [34] to 237 Mg C ha^−1^ in Cameroon [27] (AGC estimated from above ground biomass using a conversion fraction of 0.47). Differences in AGC estimates among sites and countries may be related to structural attributes, such as variable stem density (e.g. ranging from < 1000 stems ha^−1^ in Gabon South to > 35,000 stems ha^−1^ in Senegal [26] but also to different sampling approaches, including minimum tree diameter sampled [35], or the equation used to estimate tree biomass [36]. Waterlogging or salinity, which affects decomposition rates, and therefore nutrients available for plant growth, might also explain some of these differences. For example, it has previously been predicted that estuarine mangroves where there are lower salinities, usually have greater aboveground stature [28]. Human interaction and harvesting of mangrove for building poles, charcoal production and agricultural clearing also has an impact on mangrove ecosystem composition [37].

We address four major research questions: do carbon stocks differ with increasing seaward distance? Are there advantages of using 1 ha plots over smaller vegetation plots? What are the effects of using ≥ 5.0 or ≥ 10.0 cm diameter thresholds on AGC estimates? And, how do AGC and SOC compare to those reported elsewhere in Africa? We hypothesized that AGC and SOC would increase with increasing seaward distance. We also hypothesized that the effects of using ≥ 5.0 or ≥ 10.0 cm diameter thresholds on AGC estimates would be highly significant, as current mangroves ecosystems are generally characterised by having numerous small stems due to historical and ongoing human use. Through this case study, we suggest methods for future mangrove research in Africa.

## Methods

### Study area

**Fig. 1:**
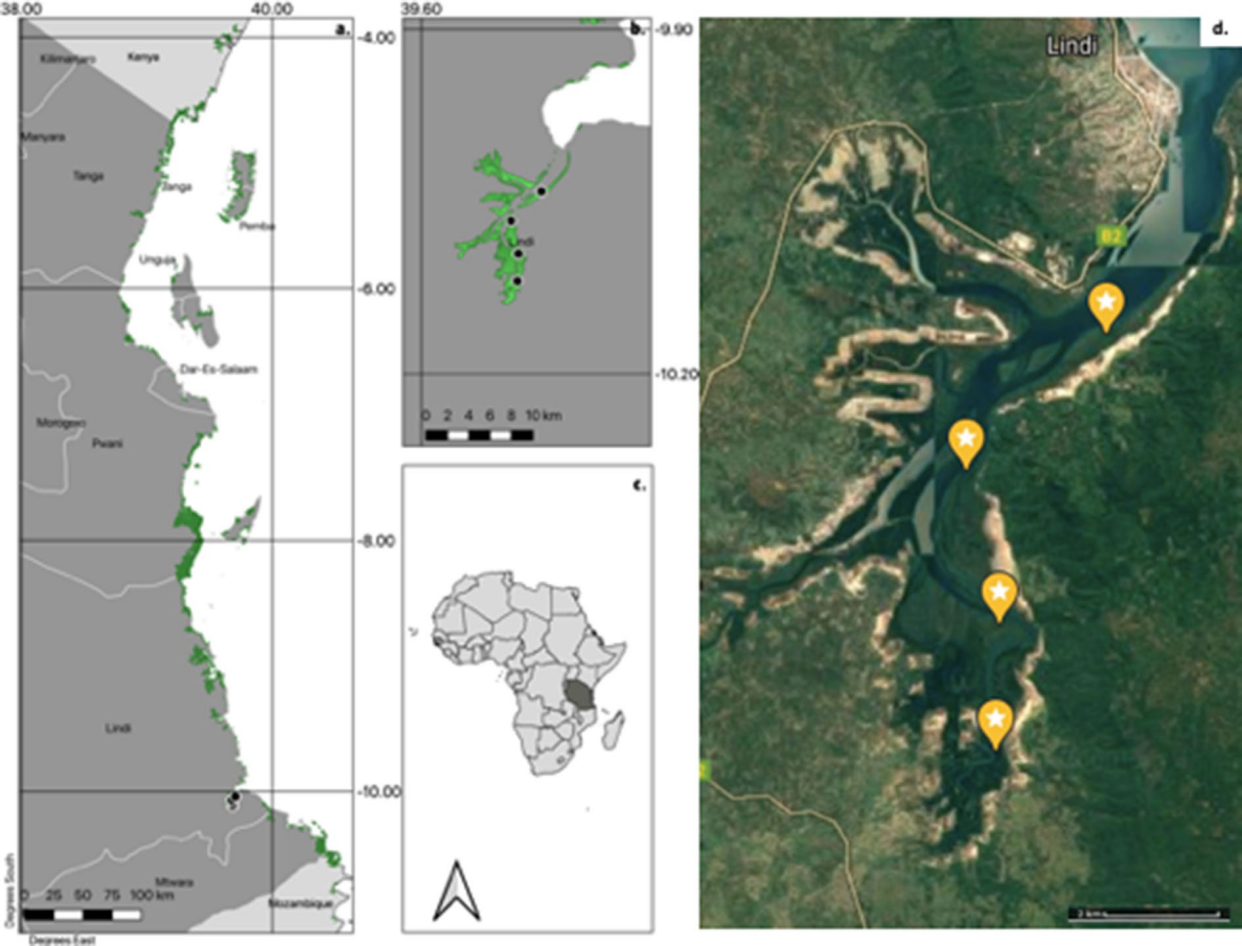
**a** Tanzanian coastline with mangroves highlighted in green; **b** Close-up of Lindi estuarine mangroves with study plots (black circles); **c** Study area in Tanzania; **d** Google satellite image of plots showing the proximity of farming; Mangrove coverage extracted from Bunting et al. [47]

This study focused on the estuarine mangroves of the Lindi region in Tanzania that currently has approximately 4500 ha of mangroves, of the 108,000 ha found in Tanzania (Fig. 1). Although this areal extent should be seen as an estimate, as the area of mangroves is not fully known and depends on how these are accounted for; for example UNEP-WCMC estimated 127,200 ha, in 2000, Francis and Bryson estimated 133,500 ha in 2001 [38, 39].The average annual temperature in Lindi is 25.7 °C, mean annual rainfall is 1200 mm year^−1^, with a rainy season that extends from October to June [40]. The coastal soils in the region consists of alluvial and sandy soils [41]. Mangroves of the study area are supported by the presence of Lukuledi, Ngurumahamba, Mtange and Mingoyo rivers mostly from Rondo catchment with the exception of the Lukuledi river which originates from Nachingwea.

Mangrove ecosystems in Tanzania have long been exploited by humans. Before and during the colonial era, poles and timber were used as building materials for boats and houses by Arabic traders [42, 43]. Mangroves continue to be exploited for firewood and poles, but large timbers requited for boats are no longer available [5, 44]. In the study area mangroves are used as source of building poles for houses, and fuelwood for lime burning to create cement [44], as well as being cleared to provide space for seaweed cultivation, illegal sand mining and salt pan construction for salt making [45, 46]. In Lindi mangrove ecosystems, construction of salt pan is mostly conducted close to the shore to allow easy and lower cost feeding of ocean water to the constructed ponds. This is associated with creation of salt pans pathways, salt storage areas and huts construction. These activities have largely been affecting the growth and stocking of mangrove species [46]. Similarly, seaweed cultivation is mostly practiced close to the shore where ocean water is permanently available. Farmers will look for open areas or clear mangrove areas, as they do for salt open construction, to establish their farms. This intensifies mangrove degradation close the shore and hence has an implication to carbon storage. This has been the opposite for illegal harvest for timber, building poles and firewood which is mostly practiced away from the shore for easy transport to the desired destinations.

### Data collection and analysis of soil samples

**Fig. 2:**
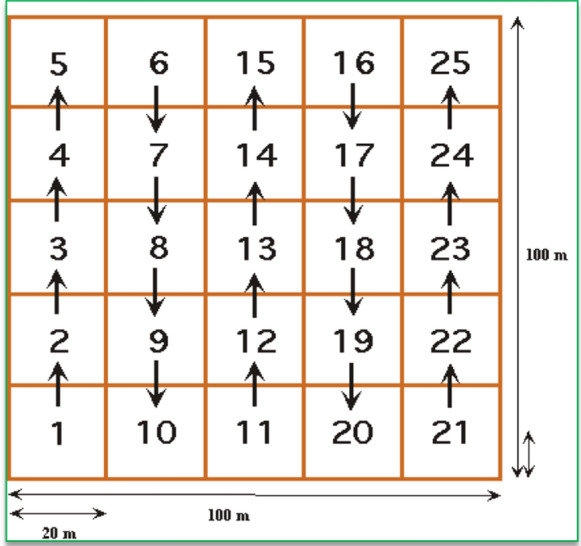
1 ha Vegetation plot, showing movement between numbered sub-plots

Four 1-ha plots were established at 4.3 km, 8.1 km, 11 km and 13.5 km along a gradient through the mangrove forest from the shore to land (Fig. 1). 1-ha plots were divided into 20 subplots of 20 × 20 m [48, 49]. These subplots were separated from one another using sisal ropes creating visual borders to avoid double measurements of stems. A systematic pattern (North–South) was then followed to measure stems in each subplot (Fig. 2). In each sub plot, the diameter at breast height (DBH; 1.3 m), the species and the height were recorded for all stems ≥ 10.0 cm. The same variables were recorded for smaller stems (≥ 5.0–9.9 cm DBH) in five subplots of 20 × 20 m (subplots 1, 5, 13, 21 and 25). Stem heights for the trees ≤ 10.0 m height were measured parallel to tree from the base to the highest point using a pole of known height [50]. Heights of the trees > 10 m were measured using a laser distance meter (Leica disto). For species which were not identified in the field, a voucher specimen was collected and taken to the National Herbarium in Arusha for further identification. In total, we sampled 2071 stems ≥ 10.0 cm and 970 stems ≥ 5–9.9 cm. Seven species of mangrove were found. Given the homogenous nature of the mangrove ecosystem this was deemed to capture the extent of any variation and provide insight into patterns of above and below ground carbon storage.

In each plot, litter biomass was recorded as follows: first, 1 m^2^ quadrats were established in the corners of subplots 1, 5, 21, 25 and at the centre of the sub plot 13. Litter materials (excluding dead wood) were collected from the five (1 m^2^) established quadrats and the total wet weight was taken. Sub-samples (50%) were taken from the whole sample, weighed before packing and transported to the lab [51, 52]. The wet combustion method was used to estimate percentage organic carbon from the dry mass of the litter [53]. A portion (50%) of the litter was oven dried to constant weight at 70.0 ^°^C to determine the dry mass [54] and grounded to fine powder for total organic carbon determination. The total organic carbon for litter was determined using the wet combustion procedure as described in Nelson and Sommers [55]. The amount of carbon in each sample was calculated as the product of percentage organic carbon and dry mass [54].

A pit of 1 m depth was dug 15 m away from each 1 ha plot. Due to the challenging environment of the mangrove ecosystem, soil pits were allocated in such a way that samples could be collected up to 1 m without water interference, through careful timing of the water tides. Soil samples were collected using a metal ring (98.12 cm volume) inserted into the sediment in a pit dug from a profile at different depths: 0–15 cm, 16–30 cm 31–60 cm and 61–100 cm. Each layer was packed separately, and soil samples were transported to the lab, air dried, grounded and passed through a 2 mm sieve to remove stones and gravel. SOC was determined based on the Walkley–Black chromic acid wet oxidation method [56] and the results were expressed as the % organic carbon. Computation of SOC density was based on soil mass per unit area obtained as the product of soil volume and soil bulk density determined from the bulk density samples in (g/cm^3^).

### AGC estimations and data analysis

Above ground biomass of all stems ≥ 5.0 cm DBH (AGB, Mg ha^−1^) was computed using different biomass equations, including generic equations derived by Komiyama et al. and Chave et al. [57, 58] (see Additional file 1). We report here the values of AGB and below ground biomass (BGB) using the multispecies equations developed by Njana et al. as these equations were derived using species from coastal regions in Tanzania, including Lindi [36]. AGC and BGC (Mg C ha^−1^) stocks were determined by using a carbon fraction of 0.47 and 0.39, respectively [59–61]. We computed AGC using stems ≥ 10.0 cm (named AGC_10_), and also using stems ≥ 5.0 cm (named AGC). We assessed the intra-plot variation in AGC by randomly sampling smaller areas (400 m^2^, 1600 m^2^, 3600 m^2^ and 6400 m^2^) of each 1 ha plot. The standard deviation relative to sampling the full 1 ha was calculated using a bootstrapping approach of 10,000 iterations. For each 1 ha plot we computed stem density (stems ha^−1^), percentage of small stems (those 5.0–9.9 cm DBH), basal area (in m^2^ ha^−1^), mean diameter (cm), mean height (m), species’ richness (number species present in the plot), species’ dominance (in terms of basal area), and species’ contribution to plot-level AGC (in percentage). Statistical analysis was carried out using R Studio (version 3.6.0). Pearson correlation coefficient was used to determine correlation between seaward distance and AGC or SOC. Paired t-tests were used to compare significant differences between AGC and AGC_10._

To compare our findings with those reported elsewhere across Africa, we carried out a literature review searching for mangrove carbon estimates across Africa.

## Results

### Above ground carbon stocks

**Fig. 3:**
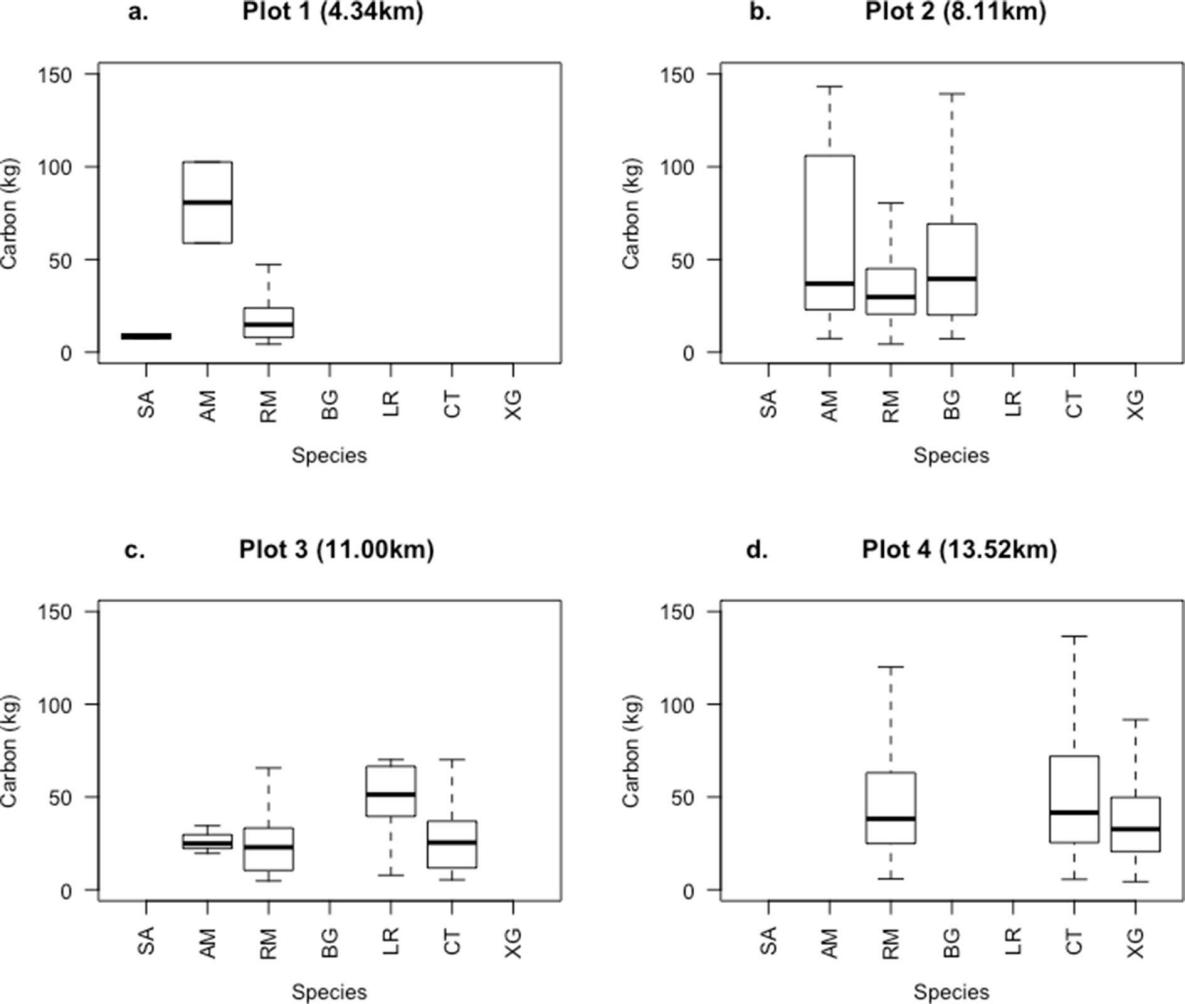
Contribution to plot level above ground carbon (AGC) (kg) of the different species found in each plot (there is increasing seaward distance from **a**–**d**). SA: *Sonneratia alba*; AM:*Avicennia marina*; RM: *Rhizophoramucronata*; BG:*Bruguieragymnorhiza*; LR: *Lumnitzera racemose*; CT: *Ceriopstagal*; XG: *Xylocarpusgranatum*

**Fig. 4:**
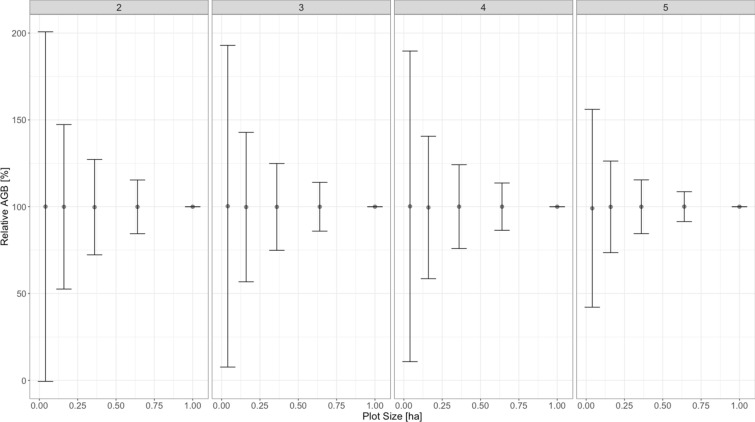
Relative AGB as a function of plots size. The error bars denote ± 1.96 × standard deviation calculated using a bootstrapping approach of 10,000 iterations of randomly sampling 400 m^2^, 1600 m^2^, 3600 m^2^ and 6400 m^2^

AGC ranged between10.9 and 54.9 Mg C ha^−1^, the mean being 26.8 Mg C ha^−1^ (Table 1). AGC was not significantly positively correlated with seaward distance (Pearson’s correlation, r^2^ = 0.4, p = 0.3, df = 2), nor was BGB (Pearson’s correlation, r^2^ = 0.4, p = 0.4, df = 2). Stem density, basal area, mean diameter and mean height increased with increasing distance to the sea (Table 1). The percentage of small stems (5.0–9.9 cm DBH) was greatest closest to shore (58%, see Table 1). Using a 5.0 cm diameter threshold significantly affects AGC estimates (paired t-test, df = 3, p-value = 0.02), although in plots 2 and 4 there was less than a 10% difference in AGC & AGC_10_. The contribution of litter to the total carbon stocks was negligible in all plots (Table 1). Species’ dominance, and contribution to AGC changed with distance from the sea (Fig. 3). There were no differences in species richness if a 5.0 cm or a 10.0 cm diameter threshold was used. Using only small plots to quantify AGB will result in higher uncertainty to represent the larger 1 ha area (Fig. 4). The trend in decreasing uncertainty with larger plot area is similar for all four plots.

**Table 1:** Distance to shore (km), stem density (stem dens/ha^−1^) of small (5–9.99 cm), all and large stems (defined as ≥ 30 cm diameter), mean diameter at breast height (DBH), mean height (H), basal area, wood mean density (WMD), Species richness (all trees), above ground carbon (AGC, all stems), AGC stems > 10 cm diameter (AGC_10_), litter carbon, below ground carbon (BGC, that stored in roots), soil organic carbon (SOC) in 0–100 cm depth

Plot	Distance to shore (km)	Stem dens. 5–9.9 cm DBH	Stem dens ≥ 10 cm	Stem dens. ≥ 30 cm DBH	Mean DBH (cm)	Mean H (m)	Basal area (m^2^ ha^−1^)	WMD (g cm^−3^)	Species richness	AGC (Mg C ha^−1^)	% difference AGC & AGC_10_	Litter (Mg C ha^−1^)	BGC (Mg C ha^−1^)	SOC (Mg C ha^−1^)	Total (Mg C ha^−1^)
1	4.34	432	304	0	8.83	5.53	4.88	0.81	3	12.75	33.12	0.05	13.71	483.63	510.14
2	8.11	171	568	1	12.53	7.62	10.06	0.8	3	28.85	7.02	0.06	24.3	327.52	380.73
3	11	160	258	0	10.52	4.39	3.97	0.82	4	10.86	15.06	0.02	10.35	241.89	263.12
4	13.52	207	941	4	13.59	7.37	18.66	0.74	3	54.9	4.24	0.03	43.3	153.73	251.96

### Soil organic carbon stocks

**Fig. 5:**
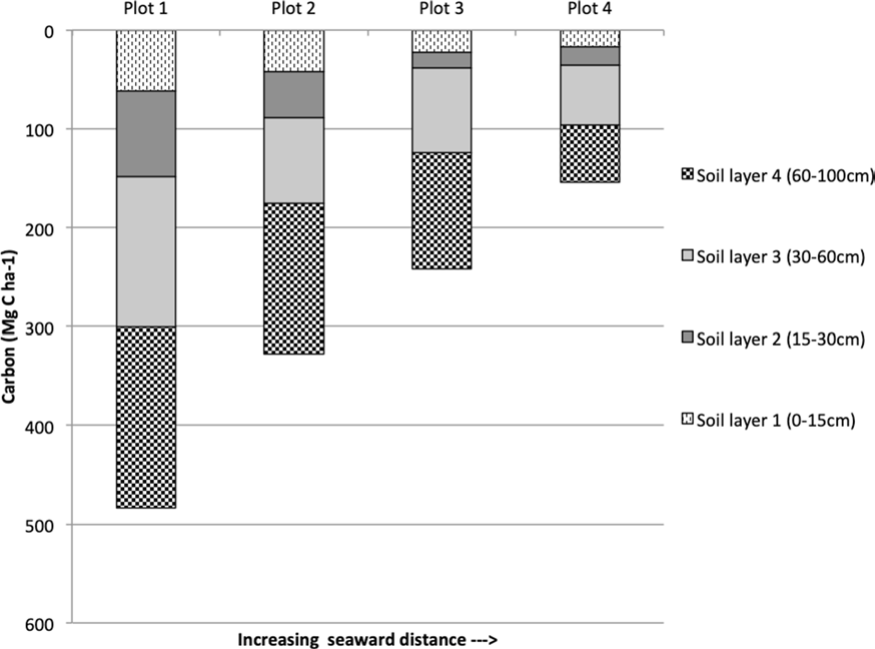
Soil carbon stocks across the four plots sampled along a seaward gradient

Soil organic carbon for 0–1 m depth ranged from 153.73 to 483.63 Mg C ha^−1^, the mean being 301.7 Mg C ha^−1^ (Fig. 5). Contrary to AGC, SOC was significantly negatively correlated with distance towards the sea (Pearson’s correlation, r^2^ = 1.0, p < 0.05, df = 2). SOC in each layer (0–15 cm, 15–30 cm, 30–60 cm and 60–1 m) decreased with increasing distance from the sea (Fig. 5).

### Carbon change with distance to the sea

Overall carbon stocks were significantly negatively correlated with distance to the sea (Pearson’s correlation, r^2^ = 0.9, p < 0.05, df = 2), with 510.1 Mg C ha^−1^ in plot 1, closest to the sea and 251.9 Mg C ha^−1^ in plot 4, furthest from the sea (Table 1).

### Comparison with other studies in Africa

The literature review of available mangrove studies across Africa is presented in Table 2.

**Table 2:** Studies reporting above ground carbon stocks (AGC) across Africa, some also reporting other carbon pools

Location	Authors	Annual rainfall	Seaward Distance reported?	Stem density (stems ha^−1^)	Basal area (m^2^ ha^−1^)	No. species	No. plots sampled	Plot size/shape	Minimum DBH	AGB Equation used (H = height used in equation)	Soil depth sampled	No. of soil samples	AGC (Mg C ha-1)	BGC (Mg C ha-1)	SOC (Mg C ha-1)	SOC method	Observations
Africa	Fatoyimbo & Simard (2013)	–	N	–	–	–	–	–	–	Mutlispecies (H) (Saenger and Snedaker, 1993)	–	–	54.52*	–	–		radar/lidar integration study
Cameroon	Ajonina et al. 2014	3000–4000 mm	N	3256	25.1	5	3 subplots × 5 PSP	20 × 10 m/1 × 1 m	>1 cm/< 1 cm	Genus specific (Ajonina et al. 2008)	1 m	60	237.35*	119.34*	–		
Democratic Republic of the Congo	Ajonina et al. 2014	772 mm	N	1267	24.5	2	3 subplots × 3 PSP	20 × 10 m/1 × 1 m	> 1 cm/< 1 cm	Genus specific (Ajonina et al. 2008)	1 m	36	192.23*	72.15*	–		
Gabon	Ajonina et al. 2014	2500–3000 mm	N	1467	24.5	8	3 subplots × 4 PSP	20 × 10 m/1 × 1 m	> 1 cm/< 1 cm	Genus specific (Ajonina et al. 2008)	1 m	48	160.27*	58.89*	–		
Gabon North	Kauffman & Bhomia, 2017	2883 mm	N	–	–	–	6 plots × 7 sites	7 m radius, 2 m radius	> 5 cm, < 5 cm	Genus specific (Fromard et al. 1998)	~1 m	42	36–380	–	345	dry combustion method (induction furnace)	
Gabon South	Kauffman & Bhomia, 2017	1818 mm	N	< 1000	–	–	6 plots × 10 sites	7 m radius, 2 m radius	> 5 cm, < 5 cm	Genus specific (Fromard et al. 1998)	~1 m	60	3–313	–	191	dry combustion method (induction furnace)	
Guinea-Bissau	Carreiras, et al. 2012	–	N	–	–	3	16	20 m, 14 m, 4 m radius concentric	> 50 cm, > 20 cm, > 5 cm	*A. germinans*, *L. racemosa* (Fromard et al.1998); R. mangle (Imbert and Rollet, 1989)	–	–	26.39*	–	–		Remote sensing study
Kenya	Gress et al. 2017	–	Y	–	–	3	77	10 × 10 m	–	–	2.5 m		–	–	1224	Oven dried	
Kenya	Cohen et al. 2013	–	N	–	–	7	337 trees	harvest data	n/a	Species specific and multispecies	–	–	35.04–96*	–	–		
Liberia	Kauffman & Bhomia, 2017	3346 mm	N	–	–	–	6 plots × 10 sites	7 m radius, 2 m radius	> 5 cm, < 5 cm	Genus specific (Fromard et al. 1998)	~1 m	60	5–162	–	342	dry combustion method (induction furnace)	
Madagascar	Jones et al. 2014	–	N	1250-5600	–	8	56	10 × 10 m and 20 × 20 m	5 cm	*A. marina* (Clough & Scott, 1989); *B. gymnorrhiza* (leaves), *C. tagal, H. littoralis* (leaves), *R. mucronata* (Comley & McGuinness, 2005); *B. gymnorrhiza* (stem, H), H. littoralis (stem, H), L. racemosa, S. alba (H), R. mucronata (stem, H), X. granatum (H) (Kauffman & Cole, 2010)	1.5 m	50	20.8–146.8	–	324-517		
Mozambique	Fatoyinbo et al. 2017			2036	–	8	25	0.0154 ha, 2 m radius	> 5 cm, < 5 cm	Njana et al. (H)	–	–	95.41*	–	–		radar/lidar integration study
Mozambique	Sitoe et al. 2014	–	N	–	–	6	55, 31 trees felled	7 m radius	> 5 cm	Multispecies (Sitoe et al. 2014)	1 m	55	28.02	25.22	160	Walkley–Black wet oxidation method	
Mozanbique	Stringer et al. 2015	1000–1400 mm	N	–	–	9	6 subplots × 12 plots	7 m, 2 m radius	> 5 cm, < 5 cm and h > 1.3 m	Multispecies (Komiyama et al. 2005, 2008)	2 m	72	25.85–113.2*	8.80-26.94*	274.6-314.1	Oven dried weight	
Republic of Congo	Ajonina et al. 2014	2500–3000 mm	N	1667	18.8	2	3 subplots × 3 PSP	20 × 10 m/1 × 1 m	> 1 × cm/< 1 cm	Genus specific (Ajonina et al. 2008)	1 m	36	117.97*	47.58*	–		
Senegal	Kauffman & Bhomia, 2017	650 mm	N	35,000	–	–	6 plots × 6 sites	7 m radius, 2 m radius	> 5 cm, < 5 cm	Genus specific (Fromard et al. 1998)	~1 m	36	11–122	–	240	dry combustion method (induction furnace)	
Tanzania	Njana et al. 2017	879–1240 mm	N	3662–4947	10.8–13.3	9	88	15 m radius concentric plots	> 1 and min height 2 m	Mulitspecies and species scpecific for Avicennia marina, Sonneratia alba and Rhizophora mucronata (Njana et al. 2016)	–	–	33.5	30.5	–		
Tanzania	Lupembe 2014	750–1250 mm	N	729	18.3	8	59 plots, 50 trees felled	20 m × 40 m	> 5 cm	Multi species (Lupembe 201)6	60 cm	50	40.5	21.08	98.57	Walkley–Black wet oxidation method	
Tanzania	This study	1200 mm	Y	717	8.82	7	5	100 m × 100 m	> 5 cm	Multipspecies (Njana et al. 2016)	1 m	20	26.84	25.79	301.69	Walkley–Black wet oxidation method	

AGC: above ground carbon; BGC: below ground carbon (e.g. roots), SOC: soil organic carbon

*Signifies that values have been computed from above ground biomass values (AGB, Mg dry mass ha^−1^) to carbon (Mg C ha^−1^) using the carbon fraction of 0.47 (Kauffman and Donato [59])

Studies on only SOC as reviewed in Twilley et al. [31] were not included in this review

## Discussion

### Above ground carbon stocks

AGC increased with increasing seaward distance, as has been reported in Qatar and Micronesia and in worldwide reviews [57, 62]. However, the correlation between AGC and seaward distance was not significant, possibly due to the low number of plots or because of the lower AGC value in plot 3 which may have experienced greater exploitation pressure. Plots closest to shore are likely to experience greater tidal inundation and salinity, lower decomposition rates, and therefore, less nutrients being available for tree growth [57]. In Lindi region, they could also suffer greater anthropogenic pressures close to the shore, for example from seaweed farming or salt pans. With increasing seaward distance, increasing mean diameter and height at plot level, stem density and basal area, and a change in species composition and abundance, translated into increased AGC.

There was a difference using a 5.0 or 10.0 cm diameter threshold, which agrees with insights from savannah ecosystems [63] but differs from flooded forest and lowland terra firma rainforests [35, 64], however the difference was particularly small when compared to SOC and the total carbon stock. Therefore, for an assessment of carbon storage in mangrove ecosystems with numerous stems > 10.0 cm, we recommend a 10.0 cm diameter threshold, which is less time-consuming during fieldwork and yields good results. Note that numerous studies across African mangrove ecosystems have used smaller diameter thresholds (Table 2). The finding that using only small plots to quantify AGB will result in higher uncertainty to represent the larger 1 ha area is concurrent with other studies [65].

Overall, our estimates of AGC (10.9–54.9 Mg C ha^−1^) are similar to those reported elsewhere in Tanzania (33.5 and 40.5 Mg C ha^−1^, Table 2), but lower than for example in the Democratic Republic of Congo [27]. This could be attributed to the combination of: (i) different methods used to sample AGC (Table 2), (ii) different environmental characteristics (e.g. ocean *vs* estuarine, different rainfall patterns, impacts of cyclones), but particularly important is likely to be (iii) the long and pervasive history of exploitation of mangroves in Tanzania [42, 43]. The satellite images of the plots (Fig. 1) indicate that there is substantial anthropogenic pressure in the area. The lower value of AGC, suggests that that current AGC quantified is significantly below the potential and could be significantly increased with appropriate control of mangrove timber harvesting and clearing combined with future management of the mangrove ecosystem that focuses on maintaining integrity of the sedimentary environment.

### Soil organic carbon stocks

SOC stocks decreased with increasing distance from the sea, which is different from studies in Micronesia, where SOC increased with increasing seaward distance because of greater soil depth [66]. Donato et al. found no change in SOC with increasing seaward distance in estuarine and oceanic mangroves in the Indo-Pacific—but all their plots were within 200 m from the seaward edge. Soil erosion and soil depth are other important factors determining SOC along seaward gradients [20]. In our study area the first plot we sampled was 4 km from the exposed shoreline where soil erosion was not an issue. Beyond the zone of soil erosion, plots closest to the shore, which experience greater tidal inundation (and salinity), have slower decomposition rates, and therefore, higher SOC stocks. Mangrove’s sediments can store high amounts of carbon due to complex root structures, high sedimentation rates and waterlogged conditions which impedes microbial degradation and slows decay [16, 25].

SOC stocks reported in this study are significantly higher than other studies in Tanzania (which only sampled 60 cm depth [67]), but they are within the range reported by other studies in Africa (Table 2). Similar to AGC, there have been variable approaches taken to sample SOC, using variable depths. Given the high amount of carbon stored in soils (as SOC), we recommend sampling mangrove sediment at least up to 1 m. Jones et al. reported about 100 Mg C ha^−1^ in the sediment layer 1–1.5 m in Madagascan mangroves [21], which suggests that sampling to greater depths would yield a true assessment of the extent of the SOC. Kauffman et al. also highlighted the importance of including soil profiles > 1 m depth in carbon stock estimates [28]. Indeed, palaeoecological investigations from Tanzanian mangrove systems clearly demonstrate that the sediment layer extends up to c. 4.0 meters [68, 69]; thus the high SOC value currently recorded down to 1 m is likely to be much greater if the full sediment system is assessed and the true value of managing the mangrove SOC realised by targeting above ground interventions to minimise any below ground disturbance. Despite its importance SOC (in addition to litter) was not included in Tanzania National FREL due to limited reliable data (URT, 2017). As SOC stocks were much greater than AGC, even further from the seashore where AGC increased, we recommend focusing on more extensive sampling of SOC so that the major repositories of carbon though soils can be quantified and fed into initiatives such as REDD+ and associated MRV systems for sustainable result-based forest conservation.

### Implications for Tanzania

In recent years there has been a drive to include carbon stocks in mangroves in reduced carbon emission targets, as they provide the potential to help mitigate and manage climate change through reducing greenhouse gas emissions [9, 70]. Managing mangroves to maximise carbon sequestration and storage that can mitigate climate change and meet national carbon emission targets first requires an accurate method of determining carbon extent [71]. We document here the importance of the mangrove above and below ground carbon store that when combined makes the ecosystem one of the most important on the planet for regulating global carbon cycles. Clearly mangrove conservation offers the potential for low cost options for reducing CO_2_ emissions [9, 72]. With the growing interest in developing and implementing market-based mechanisms such as carbon offsets and programs such as the Reduced Emissions from Deforestation and Degradation (REDD+) [70, 73], quantifying carbon stocks of mangrove forests at national, regional and continental levels is key [74], to national and local participating in climate change mitigation strategies such as REDD+ [71, 75]. Clearly, mangrove ecosystems have extensive capacity to store carbon, along with being one of the most productive ecosystems [76] in terms of net primary production. This contribution into international climate change mitigation is only one economic contribution with mangroves ecosystems supporting and providing economic security for local livelihoods in terms of fisheries support, coastline protection, pollution buffering, and water and sediment stabilisation [9, 20, 77]. Mangrove ecosystems have a long history of human exploitation and have recently undergone a 30–50% decline in area extent over the past 50 years and are expected to fully functionally disappear in under 100 years [78]. There is an urgency to assess the full role of mangrove ecosystems in climate change mitigation [7]; and ensure that the future contribution of mangroves to provide international and local ecosystem services can be maximised.

## Conclusion

This study has shown that seaward distance has an important effect on both AGC and SOC stocks in the Lindi region of Tanzania. It has also highlighted that mangrove carbon studies available for Africa do not describe type of mangrove (estuarine, oceanic), or consider seaward distance, which makes comparisons across sites challenging [20]. Although more research on the environmental factors behind seaward distance are needed (e.g. salinity, flooding tidal periodicity, nutrients and soil porosity), we highlight that seaward distance should be reported in mangrove studies in the continent. We also recommend focusing on trees > 10.0 cm diameter, and sampling soils to greater than 1 m depth which would provide a more complete assessment of the mangrove carbon store. Using large permanent sample plots, which reduce sampling uncertainties [79], and sampling tree height in the field, which is known to improve long term AGC dynamic estimates [79], are also advised.

Overall, mangroves in Lindi store a substantial amount of carbon, particularly, in the sediment. Once disturbed, SOC cannot be regained over meaningful human timescales because mangrove sediment deposits take thousands of years to form [17, 68, 69]. Because SOC is protected by the above ground vegetation, mangrove tree conservation is of key importance. The highlighted limitations of SOC and AGC limitations in mangrove ecosystem in Tanzania call for increased efforts to integrate mangroves into Tanzanian REDD+ future process such as updated Forest Reference Emission Level Assessment (FREL) [80]. Such efforts will enable Tanzania—and other African nations—to fully benefit from carbon offsetting national and international schemes.

## Supplementary information

**Additional file 1.** Allometric equations used to calculate above ground biomass (AGB) (ton h^−1^). Parameters include diameter at breast height (DBH) (cm), wood specific density (p) (g cm^−3^) and height (ht) (m)

## Data Availability

The datasets analysed during the current study will be made available through Figshare or can be available from the corresponding author on request.

## Abbreviations

AGC: Above ground carbon
BGB: Below ground biomass
DBH: Diameter at breast height
REDD+: Reducing Emissions from Deforestation and Degradation
SOC: Soil organic carbon
UNEP WCMC: United Nations Environment Programme World Conservation Monitoring Centre
UNFCCC: United Nations Framework Convention on Climate Change
