# Supplementary material for: The effects of seaward distance on above and below ground carbon stocks in estuarine mangrove ecosystems
Source: Carbon Balance Manag. 2020 Dec 7;15:27. doi: 10.1186/s13021-020-00161-4 (PMC7722422; doi:10.1186/s13021-020-00161-4)
Supplement: Supplementary file 1 — Additional file 1. Allometric equations used to calculate above ground biomass (AGB) (ton h−1). Parameters include diameter at breast height (DBH) (cm), wood specific density (p) (g cm−3) and height (ht) (m) [file 13021_2020_161_MOESM1_ESM.docx]

Additional file

Table 1 Allometric equations used to calculate above ground biomass (AGB) (ton h^-1^). Parameters include diameter at breast height (DBH)(cm), wood specific density (*p*) (g cm^-3^) and height (ht) (m)

| **Species Specific AGB** | **Equation (calculation of AGB)** | **Country** | **Reference** | Average carbon (mg C ha ^-1^) |
| --- | --- | --- | --- | --- |
| *Xylocarpus granatum* | 0.0000823*DBH^2.59^ | Australia | (Clough and Scott, 1989) | 25.19 |
| *Ceriops tagal* | ﻿ 0.000189*DBH^2.34^ | Australia | (Clough and Scott, 1989) |  |
| *Rhizophora mucronata* | ﻿ 0.00025128*DBH﻿^2.26026^ | Tanzania | (Njana, 2015) |  |
| *Bruguiera gymnorhiza* | ﻿ 0.000186*DBH^2.31^ | Australia | (Clough and Scott, 1989) |  |
| *Avicennia marina* | ﻿ 0.00025128*DBH﻿^2.24351^ | Tanzania | (Njana *et al.*, 2015) |  |
| *Sonneratia alba* | ﻿ 0.00025128*DBH﻿^2.21727^ | Tanzania | (Njana *et al.*, 2015) |  |
| *Lumnitzera racemosa* | 0.000184*DBH^2.384^ | Indonesia | (Kangkuso *et al.*, 2016) |  |
| **Generic AGB equations** | 0.00025128*DBH^2.24034^ | Tanzania | (Njana *et al.*, 2015) | 25.16 |
|  | ﻿ 0.000716*DBH^2.0037^ | Tanzania (Rufiji) | (Lupembe, 2014) | 38.20 |
|  | ﻿ 0.000251**p**DBH^2.46^ |  | (Komiyama et al. 2008) | 35.50 |
|  | ﻿ 0.000112*(*p**DBH^2^*ht)^0.916^ |  | (Chave et al. 2014) | 19.08 |
| **BGB Equation** | 1.4204*(DBH^1.59666^) | Tanzania | (Njana *et al.*, 2015) |  |

Figure 1 The total above ground carbon (AGC, Mg ha^-1^) across five permanent sample plots (PSP) calculated using five different allometric equations. See Table 2 for species-specific equations
